# NeuroLens: organ localization using natural language commands for anatomical recognition in surgical training

**DOI:** 10.1007/s11548-025-03463-5

**Published:** 2025-06-24

**Authors:** Nevin M. Matasyoh, Daniel Delev, Waseem Masalha, Franziska Mathis-Ullrich, Ramy A. Zeineldin

**Affiliations:** 1 Surgical Planning and Robotic Cognition Laboratory (SPARC), Department of Artificial Intelligence in Biomedical Engineering, Friedrich-Alexander-University Erlangen-Nuremberg, Nürnberger Str. 74, 91052 Erlangen, Bavaria Germany; 2 Neurosurgical Clinic, University Hospital Erlangen, Schwabachanlage 6, 91054 Erlangen, Bavaria Germany; 3 Faculty of Electronic Engineering (FEE), Menoufia University, El-Gaish St, Menouf, 32952 Menoufia Egypt

**Keywords:** Anatomical localization, Multimodal learning, Surgical training, System usability scale (SUS)

## Abstract

**Purpose:**

This study introduces NeuroLens, a multimodal system designed to enhance anatomical recognition by integrating video with textual and voice inputs. It aims to provide an interactive learning platform for surgical trainees.

**Methods:**

NeuroLens employs a multimodal deep learning localization model trained on an Endoscopic Third Ventriculostomy dataset. It processes neuroendoscopic videos with textual or voice descriptions to identify and localize anatomical structures, displaying them as labeled bounding boxes. Usability was evaluated through a questionnaire by five participants, including surgical students and practicing surgeons. The questionnaire included both quantitative and qualitative sections. The quantitative part covered the System Usability Scale (SUS) and assessments of system appearance, functionality, and overall usability, while the qualitative section gathered user feedback and improvement suggestions. The localization model’s performance was assessed using accuracy and mean Intersection over Union (mIoU) metrics.

**Results:**

The system demonstrates strong usability, with an average SUS score of 71.5, exceeding the threshold for acceptable usability. The localization achieves a predicted class accuracy of 100%, a localization accuracy of 79.69%, and a mIoU of 67.10%. Participant feedback highlights the intuitive design, organization, and responsiveness while suggesting enhancements like 3D visualization.

**Conclusion:**

NeuroLens integrates multimodal inputs for accurate anatomical detection and localization, addressing limitations of traditional training. Its strong usability and technical performance make it a valuable tool for enhancing anatomical learning in surgical training. While NeuroLens shows strong usability and performance, its small sample size limits generalizability. Further evaluation with more students and enhancements like 3D visualization will strengthen its effectiveness.

## Introduction

Surgical training is a fundamental aspect of surgical practice, ensuring that students and junior surgeons develop the technical skills and competencies required for safe and effective surgical procedures. Effective training largely depends on regular participation in surgical procedures, which is often not viable due to conflicts with the need for optimized workflow and shorter procedure durations. Outside the operating room, training has traditionally relied on structured courses, academic literature, and multimedia resources [1]. Consequently, there is a growing demand for innovative training approaches, powered by advanced technologies, to reduce over-reliance on direct participation in live surgeries. Videos have emerged as a valuable tool to address some of these gaps, providing trainees with greater exposure to diverse and complex operative scenarios while also serving as a method for assessing technical skills and clinical competency. They are widely used to demonstrate anatomy and surgical techniques across various training levels [2]. However, creating effective training videos may require annotation, which demands time, expertize, and resources that are typically scarce in busy clinical settings [3].

To overcome these challenges, advanced technologies such as artificial intelligence (AI) are increasingly being adopted to streamline medical processes, including surgical training. AI-powered tools offer solutions that go beyond the capabilities of traditional videos, enabling automated annotation, personalized feedback, and deeper insights into surgical techniques [3–6]. Natural Language Processing (NLP), a subfield of AI, focuses on developing models that can understand, interpret, and generate human language. It is classified into Natural Language Understanding (NLU) and Natural Language Generation (NLG). NLU is responsible for extracting meaning from text or speech using techniques such as tokenization, syntactic parsing, and entity recognition, allowing machines to comprehend and process human language [7]. In contrast, NLG is a complementary process that transforms structured or unstructured data into coherent text or speech, enabling machines to generate human-like responses [7].

NLP has been employed in diverse applications such as medical report generation, where it automates the creation of structured reports from unstructured clinical data. For instance, NLP techniques have been used to extract key information from CT liver tumor reports, transforming them into structured formats to improve disease assessment and treatment recommendations [8]. Similarly, an adversarial reinforcement learning-based approach has been developed for generating chest X-ray reports [9]. In clinical decision support, NLP-based word embeddings and machine learning techniques have been applied to develop a recommendation system for breast cancer diagnosis, aiding physicians in decision-making [10]. Additionally, NLP has been used for workflow automation, with a multimodal large language model (MLLM)-based framework proposed to enhance surgical understanding by performing real-time scene analysis, surgical instrument detection, and segmentation [11].

Similarly, Computer Vision, driven by deep learning, has made significant strides in medical imaging, real-time image analysis during surgeries, and robotic-assisted procedures. It integrates concepts from image processing, pattern recognition, artificial intelligence, and computer graphics to emulate aspects of human visual perception [12]. In medical imaging, multimodal deep learning techniques have been applied for tumor segmentation, where fusing different imaging modalities such as magnetic resonance imaging (MRI), computed tomography (CT), ultrasound, and positron emission tomography (PET) improve lesion detection and classification accuracy [13, 14]. Additionally, point cloud registration has been explored to align 3D medical images with augmented reality (AR)-captured point clouds, enhancing image-to-patient registration in AR-guided surgery [15]. In real-time surgical image analysis, deep learning-based detection models, including Faster-RCNN and YOLOv4, have been employed for surgical instrument detection and tracking in laparoscopic surgery [16]. In robotic-assisted surgery, advanced segmentation models such as SAMSurg [17], an adaptation of the Segment Anything Model (SAM), have been developed to segment surgical instruments across diverse domains.

Building on advances in NLP and Computer Vision, multimodal deep learning integrates sound, textual, and visual data to create enriched, context-aware representations [3]. It enables applications such as image captioning [18, 19], where textual descriptions are generated from visual inputs, and visual question answering (VQA) [20, 21], where models respond to textual queries about an image. This integration not only enhances the accuracy of such systems but also makes them robust to missing or ambiguous data in one modality, as the other can compensate to a certain extent. Thus, multimodal deep learning represents a significant leap toward building systems that more closely mimic human-like understanding and interaction with the world.

In this work, we harness the strengths of multimodal learning to develop a system that addresses the challenges of surgical training, specifically in anatomical recognition. Mastery of anatomy is critical in this field, as even minor inaccuracies in identifying and understanding these structures can lead to severe complications, making it essential for surgical trainees to have robust educational tools to build their skills. However, traditional training methods often fail to provide an interactive, context-rich environment that effectively supports anatomical understanding.

To address these challenges, we propose NeuroLens, an innovative multimodal system to enhance surgical training. NeuroLens integrates neuroendoscopic surgical videos with textual or audio descriptions to identify and highlight frames containing the described organ, annotating them with bounding boxes. Accessible through an intuitive user interface, NeuroLens enables students and young attending surgeons to interact dynamically with the videos. By combining multimodal inputs and real-time interaction, NeuroLens equips trainees with the tool they need to better understand the complex anatomy involved in neuroendoscopy, ultimately improving their surgical proficiency.

**Fig. 1:**
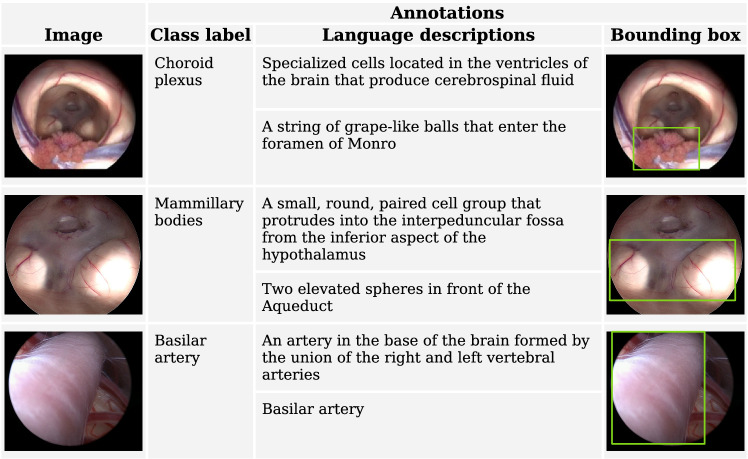
Representative sample dataset with annotated medical images, including organ labels, language descriptions, and bounding box annotations for anatomical structures

The key contribution of this work is the development of NeuroLens as a specialized tool designed to enhance anatomical structure recognition in surgical training. It addresses the limitations of traditional training by integrating video with textual or auditory inputs for precise anatomical detection and localization. Additionally, NeuroLens serves as a user-centric learning platform, allowing trainees to engage in self-directed learning in real-time. To assess the system’s effectiveness, we conducted an evaluation involving both student and practicing surgeons, ensuring a thorough assessment of its practical usability in clinical settings.

## Methods

### Dataset

The Endoscopic Third Ventriculostomy (ETV) dataset for visual grounding was used to train and evaluate the localization model [3, 22]. This dataset, curated from 29 recorded ETV procedures, comprises 1,718 images with 4,013 annotated intracranial structures and organs, each labeled with bounding boxes and accompanied by at least three language descriptions of varying levels of technical detail for each structure (refer to Fig. 1). Of these, 1,645 images are allocated for training and validation, while the remaining 73 images are reserved for testing. Additionally, two other ETV videos were utilized to evaluate the performance of the overall system.

### Overview of neuroLens architecture

**Fig. 2:**
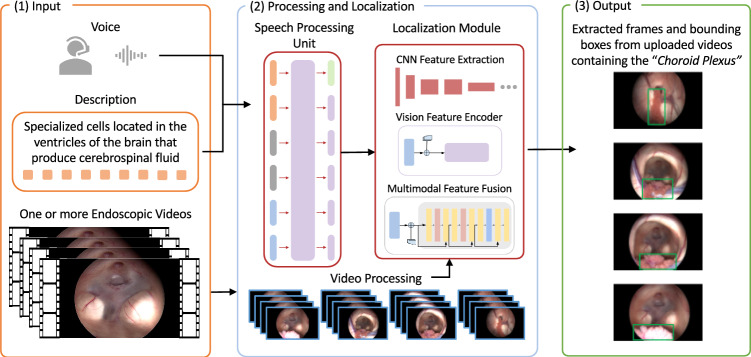
A high-level overview of NeuroLens system architecture including (1) Input data, (2) video and speech processing and localization model, and (3) extracted frame outputs with the bounding boxes for the selected label

Figure 2 presents the proposed NeuroLens system which consists of three main components: the user interface, the speech processing unit, and the localization model. The interface facilitates communication between the user and the system. The speech processing unit transcribes and refines the language description input, ensuring accurate interpretation. The localization model processes this input to identify and locate the described organ, providing the coordinates of a bounding box along with a predicted label. These coordinates are mapped to the corresponding video frames and returned to the user, together with the predicted label, through the interface.

#### User interface

**Fig. 3:**
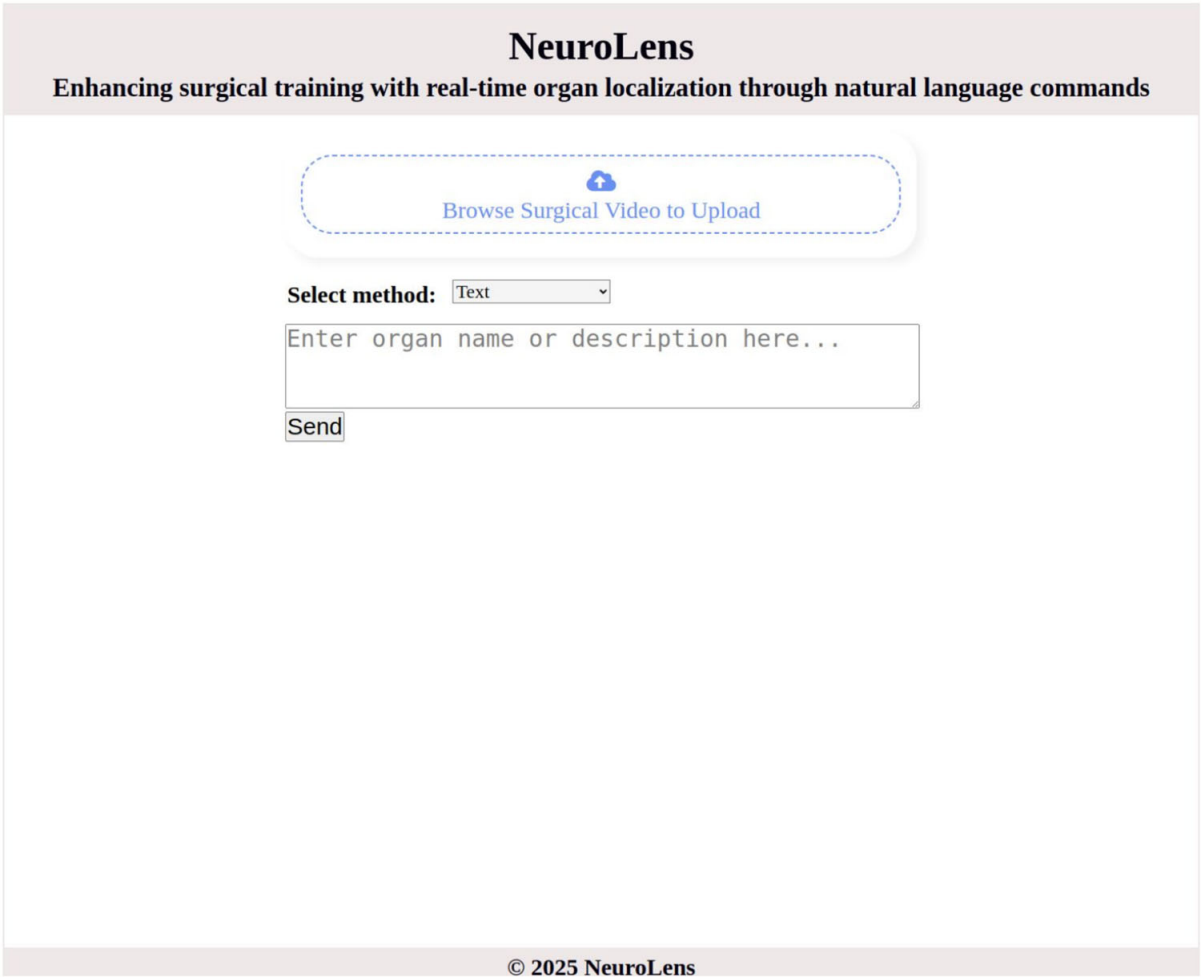
Graphical user interface of the NeuroLens system. The interface includes a video upload section for adding videos and an input selection area for choosing between text or voice inputs, which can then be submitted for processing

The NeuroLens system features an intuitive interface (see Fig. 3) that simplifies interaction with surgical videos using language descriptions. The workflow begins with the upload of one or more surgical videos, which serve as the visual input. Users can then provide a description of the anatomical structure to be identified using either text or voice input. The natural language input accommodates varying levels of specificity, ranging from simple organ names such as "choroid plexus" to detailed phrases or descriptions such as "specialized cells located in the ventricles of the brain that produce cerebrospinal fluid." This dual-input functionality enhances accessibility and flexibility, allowing interactions to suit the diverse preferences of trainees.

Upon input submission, the system processes the video and description to generate visual feedback. The video data is analyzed, and the described anatomical structure is identified and localized in the video frames using the localization model. The localized structure is highlighted with bounding boxes, providing a prompt visual representation. The interface also supports iterative interaction, allowing users to input new descriptions for the same video or refine previous inputs to explore additional anatomical features. This dynamic feedback loop fosters a seamless and engaging user experience, promoting both precision and exploration in anatomical education.

#### Speech processing unit

**Fig. 4:**
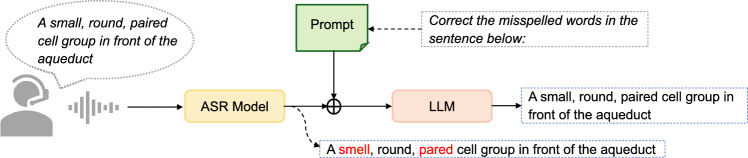
Speech processing module. The ASR model used is Whisper-medium, and the LLM is GPT$-$3.5. Words transcribed incorrectly by the ASR are highlighted in red

We adopt the speech processing technique proposed in [23], utilizing the Whisper-medium [24] and GPT-3 [25] models from OpenAI to transcribe and refine the user’s voice input (Fig. 4). The Whisper model, based on the encoder-decoder architecture of the Transformer, is a general-purpose automatic speech recognition (ASR) tool capable of converting spoken language into written text. Trained on a vast and diverse audio dataset, Whisper excels in multilingual speech recognition, making it highly robust against varying accents, background noise, and linguistic nuances [23].

Complementing Whisper, GPT-3 is a powerful large language model (LLM) designed to generate text that closely mimics human language. With its ability to understand and process nuanced language, GPT-3 is widely used in applications ranging from creative writing, such as drafting essays and stories, to functional tasks like language translation, document summarization, and powering conversational agents. In this context, GPT-3 ensures that transcriptions are accurate, clear, and semantically aligned with the user’s intent.

The audio description provided through the interface is initially transcribed into text using the Whisper-medium model. This transcription is then refined by GPT-3 to correct errors, enhance clarity, and ensure consistency. The refined text is subsequently passed into the localization model, where it is used to identify and locate the described structure or organ within the surgical video frames.

#### Localization model

**Fig. 5:**
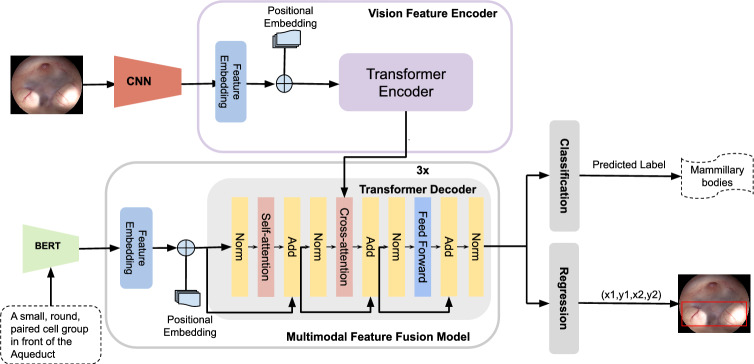
Architecture of the localization model. The model includes a vision feature encoder for processing visual inputs, a multimodal feature fusion module that processes language inputs and integrates visual and language features, and classification and regression heads for label prediction and bounding box regression, respectively

Structure localization was performed based on our previous anatomical structure localization framework [3], which we adapted to include a classification head for organ detection. The localization model utilizes the encoder-decoder architecture of conventional Transformer (refer to Fig. 5). In this design, the encoder processes the visual input and extracts meaningful features using a hybrid convolutional neural network (CNN) and a Transformer. Then, the decoder handles the language description and serves as a cross-modal feature fusion module, integrating the image features from the encoder with the language description.

**Visual input processing: **The visual branch utilizes the ResNet backbone [26], as CNN, and the Transformer encoder to generate image feature mappings. The video input is divided into individual frames, and each frame is processed sequentially. For each frame, the ResNet backbone generates a low-level image feature map, which is then flattened into a sequence of feature embeddings to meet the input requirements of the Transformer encoder. The flattened feature sequence is passed into the Transformer encoder, which extracts advanced image features. These advanced features are later fused with language features in the Transformer decoder.

**Language input processing: **The language branch employs the DistilBERT tokenizer and model [27] as the backbone for processing the language description. The input description is tokenized using the DistilBERT tokenizer and then passed through the DistilBERT model to generate advanced language features. These features are fused with the advanced image features from the Transformer encoder through the cross-attention layer of the Transformer decoder. The resulting multimodal feature map is used to predict the four coordinates of the bounding box and class label, providing the final output.

**Table 1:** SUS questionnaire. Rating on a scale of 1 (Strongly Disagree) to 5 (Strongly Agree)

Label	Question	1	2	3	4	5
Q1	I think that I would like to use this system frequently	$\square$	$\square$	$\square$	$\square$	$\square$
Q2	I found the system unnecessarily complex	$\square$	$\square$	$\square$	$\square$	$\square$
Q3	I thought the system was easy to use	$\square$	$\square$	$\square$	$\square$	$\square$
Q4	I think that I would need the support of a technical person to be able to use this system	$\square$	$\square$	$\square$	$\square$	$\square$
Q5	I found the various functions in this system were well integrated	$\square$	$\square$	$\square$	$\square$	$\square$
Q6	I thought there was too much inconsistency in this system	$\square$	$\square$	$\square$	$\square$	$\square$
Q7	I would imagine that most people would learn to use this system very quickly	$\square$	$\square$	$\square$	$\square$	$\square$
Q8	I found the system very cumbersome to use	$\square$	$\square$	$\square$	$\square$	$\square$
Q9	I felt very confident using the system	$\square$	$\square$	$\square$	$\square$	$\square$
Q10	I needed to learn a lot of things before I could get going with this system	$\square$	$\square$	$\square$	$\square$	$\square$

## Experimental setup

### Implementation details

**Training: **The localization model is trained and evaluated on a dataset of 1,718 annotated images. Each input image is resized to 256 $\times$ 256 pixels while maintaining its original aspect ratio, and the maximum length of the language descriptions is restricted to 64 tokens. To improve variability and robustness, various augmentation techniques are applied to both the images and language descriptions during training. The model is trained end-to-end using the AdamW optimizer [28] and supervised using a combination of Distance IoU (DIoU) loss [29] and smooth L1 [30] loss functions.

**Inputs: **NeuroLens accepts an input of one or more videos along with a language description in text or voice prompts. The videos are split into individual frames, which are then resized to meet the input requirements of the localization model. Similarly, the language description is tokenized and resized to match the model’s input specifications.

**Output: **NeuroLens returns video frames containing the described organ, localized with bounding boxes. The bounding boxes predicted by the localization model are mapped back to their respective video frames and returned to the user. Additionally, the output includes the name of the described organ.

### Evaluation metrics

**Usability: **Usability was evaluated using both quantitative and qualitative methods. The primary quantitative method employed was the System Usability Scale (SUS), a widely recognized and reliable survey tool used to assess the usability of a product. SUS is a standardized instrument designed to quantitatively evaluate factors such as intuitiveness, user satisfaction, and perceived complexity [31]. It consists of ten questions presented on a five-point Likert scale (i.e., strongly disagree, disagree, undecided, agree, and strongly agree) (Refer to Table 1). Participants were tasked to indicate their level of agreement with each statement on the scale.

In addition to SUS, the evaluation included an assessment of the general aspects of NeuroLens, focusing on its overall appearance and functionality (Refer to Table 2). Its adaptability was also analyzed by evaluating its ability to process diverse language inputs. These metrics provided a comprehensive understanding of the system’s usability and effectiveness.

**Table 2:** Assessment of the overall appearance and functionality. Rating on a scale of 1 (Strongly Disagree) to 5 (Strongly Agree)

Question	1	2	3	4	5
**Overall appearance**					
The interface is intuitive and easy to use	$\square$	$\square$	$\square$	$\square$	$\square$
The layout is organized and logical	$\square$	$\square$	$\square$	$\square$	$\square$
The design is visually appealing	$\square$	$\square$	$\square$	$\square$	$\square$
The interface is responsive and fast enough	$\square$	$\square$	$\square$	$\square$	$\square$
**Functionality**					
The system accurately identifies organs/structures	$\square$	$\square$	$\square$	$\square$	$\square$
The bounding boxes are correctly positioned	$\square$	$\square$	$\square$	$\square$	$\square$
The system responds appropriately to the input descriptions	$\square$	$\square$	$\square$	$\square$	$\square$
The text input works well	$\square$	$\square$	$\square$	$\square$	$\square$
The voice input works well	$\square$	$\square$	$\square$	$\square$	$\square$
The process is fast and efficient	$\square$	$\square$	$\square$	$\square$	$\square$

**Localization model performance: **The accuracy of the localization model was assessed using two key metrics: Accuracy and mean Intersection over Union (mIoU). Accuracy is the percentage of the correct predictions while mIoU is the average IoU of all the predicted bounding boxes.

### System evaluation

The NeuroLens evaluation was conducted using a two-fold approach, focusing on the performance of the localization model and a usability study. First, the localization model was evaluated using mIoU and accuracy. For the usability study, five participants from the Department of Neurosurgery at University Hospital Erlangen, Germany, were included to capture diverse perspectives on the system’s functionality and practicality. The group comprised one surgical student, two surgical residents, and two practicing surgeons, with experience levels ranging from 0 to 10 years.

Initially, participants were provided with an overview of NeuroLens features and instructions on interaction with its various modules. Then, they assessed how NeuroLens can localize anatomical structures in three surgical videos based on given language descriptions (Refer to descriptions in Fig. 1). It is worth mentioning that these descriptions were given as a reference, but the evaluators tested different prompts as well.

After completing their interaction with the system, participants filled out a questionnaire designed to evaluate multiple aspects of its usability and effectiveness. The questionnaire included a quantitative section assessing general aspects of the system, a SUS section to evaluate user satisfaction and system intuitiveness, and an open-ended feedback section that allowed participants to express their thoughts beyond predefined answer choices, enabling deeper insights into usability challenges, feature preferences, and areas for improvement.

## Results and discussion

Table 3 presents the individual responses from the five participants to the SUS assessment. The calculated SUS scores ranged from 47.5 to 92.5, with an overall average score of 71.5. Among the participants, three rated the system above the widely accepted SUS threshold of 68, indicating a good level of usability and alignment with established benchmarks for effective systems. However, two participants rated the system below this threshold, with the lowest score recorded at 47.5. This variability in scores suggests a generally favorable perception of usability among most participants, while also highlighting areas requiring improvement to address the concerns of less-satisfied users.

In addition to the SUS scores, NeuroLens was evaluated for its overall appearance and functionality. Four participants either strongly agreed or agreed that the system was intuitive, well-organized, visually appealing, and responsive. These attributes are critical for designing an interactive system that fosters ease of use and engagement. However, one participant raised concerns about its accuracy, potentially indicating variability in its performance. Addressing these issues will be essential for maintaining user trust and ensuring the reliability of hands-on surgical training.

Participants also provided qualitative feedback that highlights opportunities for future enhancements. P3 suggested incorporating 3D visual feedback instead of bounding boxes on 2D images. This recommendation shows the need for more advanced visualization techniques to provide a clearer and more interactive understanding of complex anatomical structures. A 3D representation could better mimic the spatial depth encountered in actual neuroendoscopic procedures, offering trainees a more realistic learning experience. P4 reported difficulty understanding the purpose of the study, which may have resulted from miscommunication or unclear instructions during the system evaluation. This feedback emphasizes the importance of providing comprehensive onboarding materials and clear guidance to ensure that users fully understand the system’s objectives and functionalities.

**Table 3:** Participant Responses to SUS questionnaire and the calculated SUS scores. SUS was calculated individually per participant based on their responses

Participant	Role	Q1	Q2	Q3	Q4	Q5	Q6	Q7	Q8	Q9	Q10	Scores
P1	Surgical student	4	1	5	1	5	2	5	2	5	2	90.5
P2	Surgical resident	4	3	3	4	4	4	4	4	3	4	47.5
P3	practicing surgeon	3	2	4	2	4	2	4	3	4	2	70.0
P4	practicing surgeon	3	3	2	5	5	3	5	4	4	1	57.5
P5	Surgical resident	3	1	5	1	5	1	5	1	4	1	92.5

**Fig. 6:**
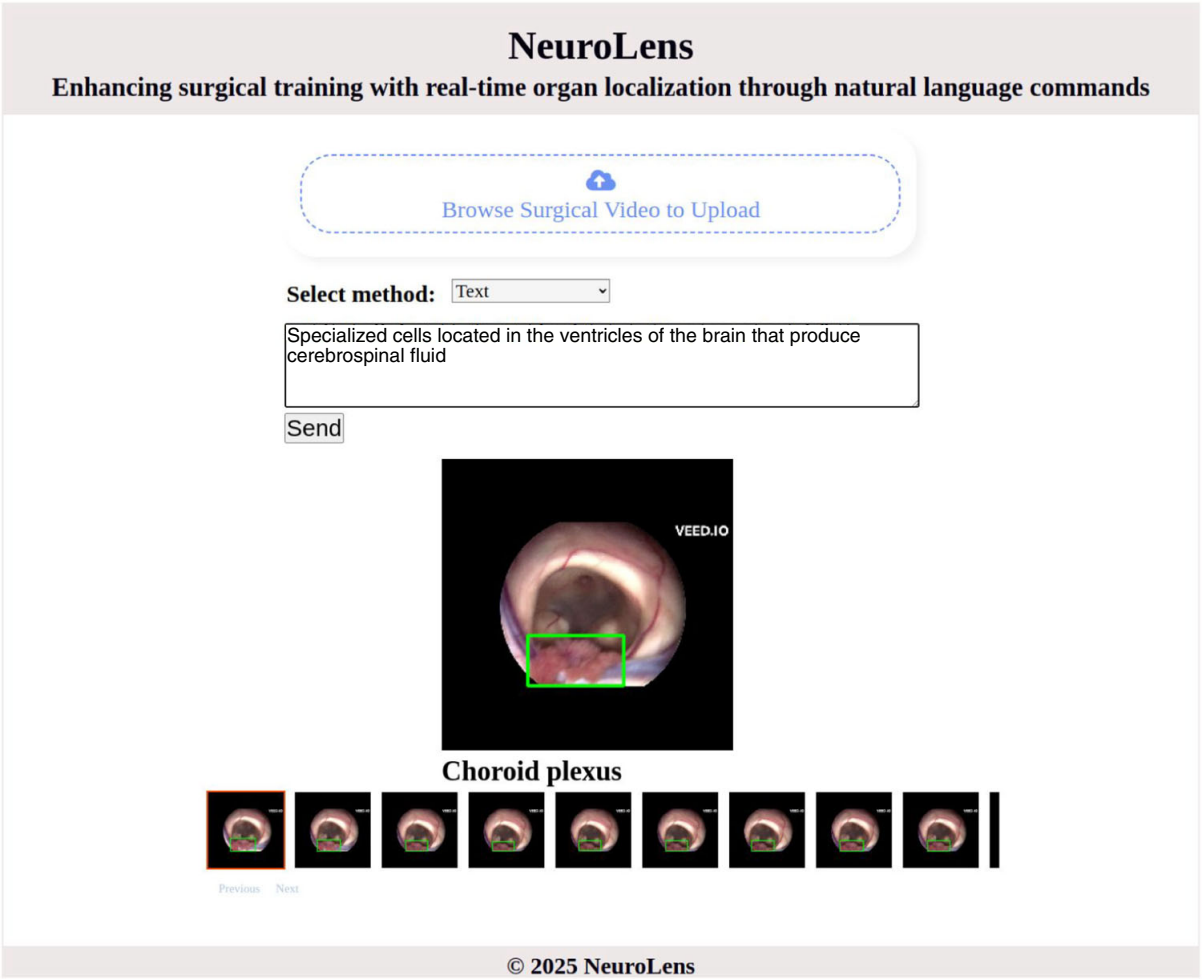
Visualization of NeuroLens outputs, showing video frames with a bounding box around the identified organ and its name

The technical performance of NeuroLens was further assessed through the effectiveness of the localization model on the test dataset. It is worth noting that the model achieved a predicted label accuracy of 100%, demonstrating its ability to precisely identify queried structures. Furthermore, the localization accuracy of 79.69% and a mean Intersection over Union (mIoU) of 67.10% highlight its effectiveness in identifying and localizing anatomical regions of interest. The qualitative visualizations of NeuroLens outputs further support these findings, demonstrating its ability to accurately identify and localize anatomical structures (Refer to Fig. 6). While these results are promising, the mIoU metric indicates room for improvement in refining the precision of bounding box overlaps. Such enhancements could significantly improve the quality of visual feedback, making the system even more effective in supporting surgical training.

Overall, these findings demonstrate that NeuroLens has strong potential to enhance surgical training by complementing traditional methods with interactive and accurate tools. Its usability, coupled with good technical performance in classification and localization, makes it a valuable resource for surgical trainees. NeuroLens is particularly beneficial for early-stage trainees, providing an interactive platform to support anatomical structure recognition. This study serves as a preliminary evaluation of NeuroLens, focusing primarily on system usability and feasibility. Future work will focus on incorporating 3D visualization to improve the clarity and depth perception of anatomical structures. We also intend to conduct a larger-scale evaluation with a broader cohort of students to assess the system’s real-world application and its impact on learning outcomes. By incorporating these improvements, NeuroLens could provide an even more engaging and effective learning experience, helping trainees build essential anatomical knowledge before transitioning to hands-on surgical practice.

## Conclusion

In this work, we developed NeuroLens, a multimodal system designed to enhance surgical training, specifically in anatomical recognition, by integrating video with textual and voice inputs for accurate anatomical detection and localization. Through rigorous experiments, NeuroLens demonstrated strong usability and technical performance. Participant feedback highlighted its intuitive design and functionality, while also identifying opportunities for improvement, such as incorporating 3D visualization.
