# Correction: NeuroLens: organ localization using natural language commands for anatomical recognition in surgical training

**DOI:** 10.1007/s11548-025-03519-6

**Published:** 2025-10-15

**Authors:** Nevin M. Matasyoh, Daniel Delev, Waseem Masalha, Franziska Mathis-Ullrich, Ramy A. Zeineldin

**Affiliations:** 1 Surgical Planning and Robotic Cognition Laboratory (SPARC), Department of Artificial Intelligence in Biomedical Engineering, Friedrich-Alexander-University Erlangen-Nuremberg, Nürnberger Str. 74, 91052 Erlangen, Bavaria Germany; 2 Neurosurgical Clinic, University Hospital Erlangen, Schwabachanlage 6, 91054 Erlangen, Bavaria Germany; 3 Faculty of Electronic Engineering (FEE), Menoufia University, El-Gaish St, Menouf, 32952 Menoufia Egypt

**Correction to: International Journal of Computer Assisted Radiology and Surgery (2025) 20:1623–1632** 10.1007/s11548-025-03463-5

In the original version of this article, in Fig. 1, the images for the “Choroid plexus” row are missing in both the Image and Bounding box columns.

The Fig. 1, which previously appeared as

**Figure:**
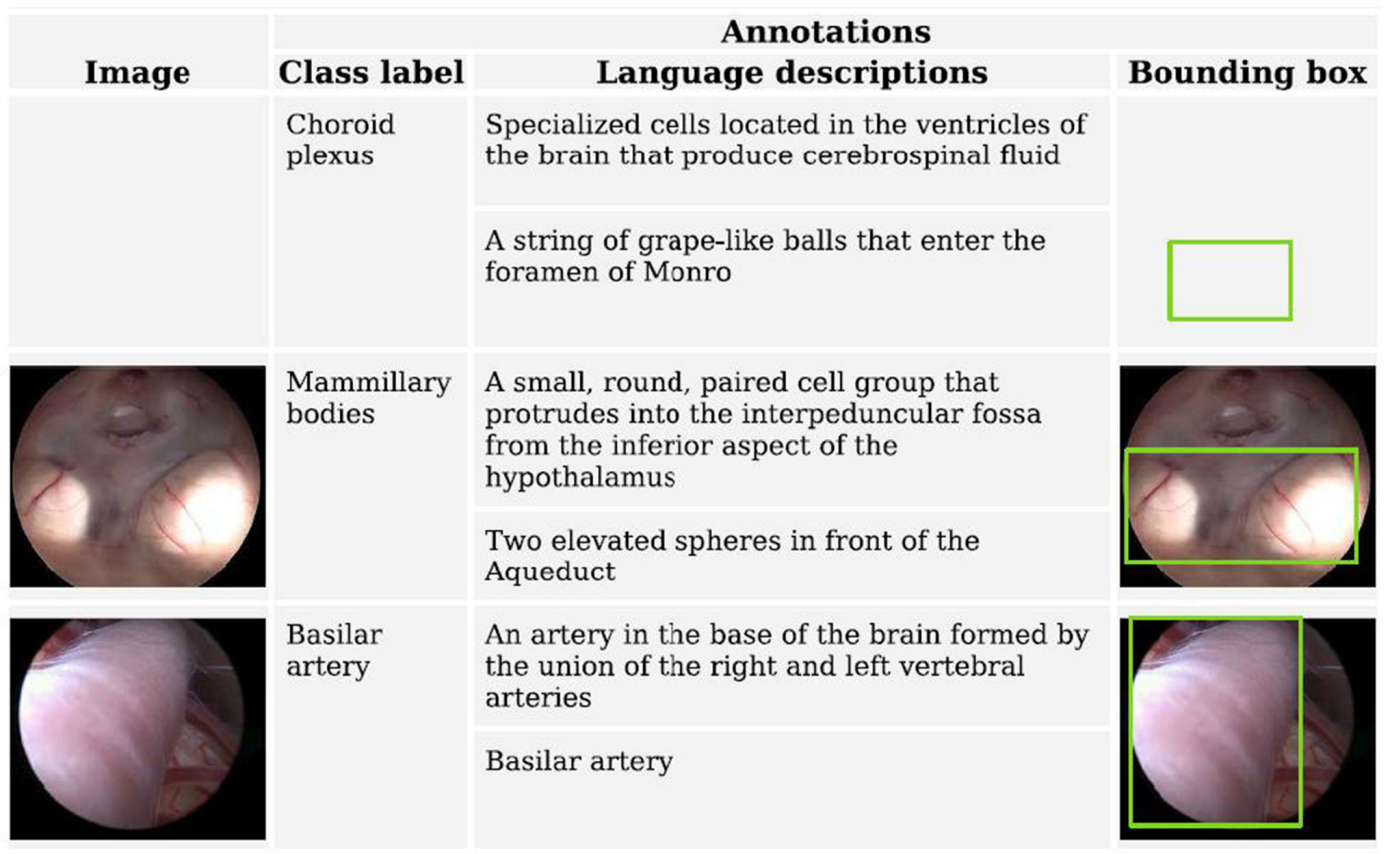
**Fig. 1** Representative sample dataset with annotated medical images, including organ labels, language descriptions, and bounding box annotations for anatomical structures

but should have appeared as shown below.

**Figure:**
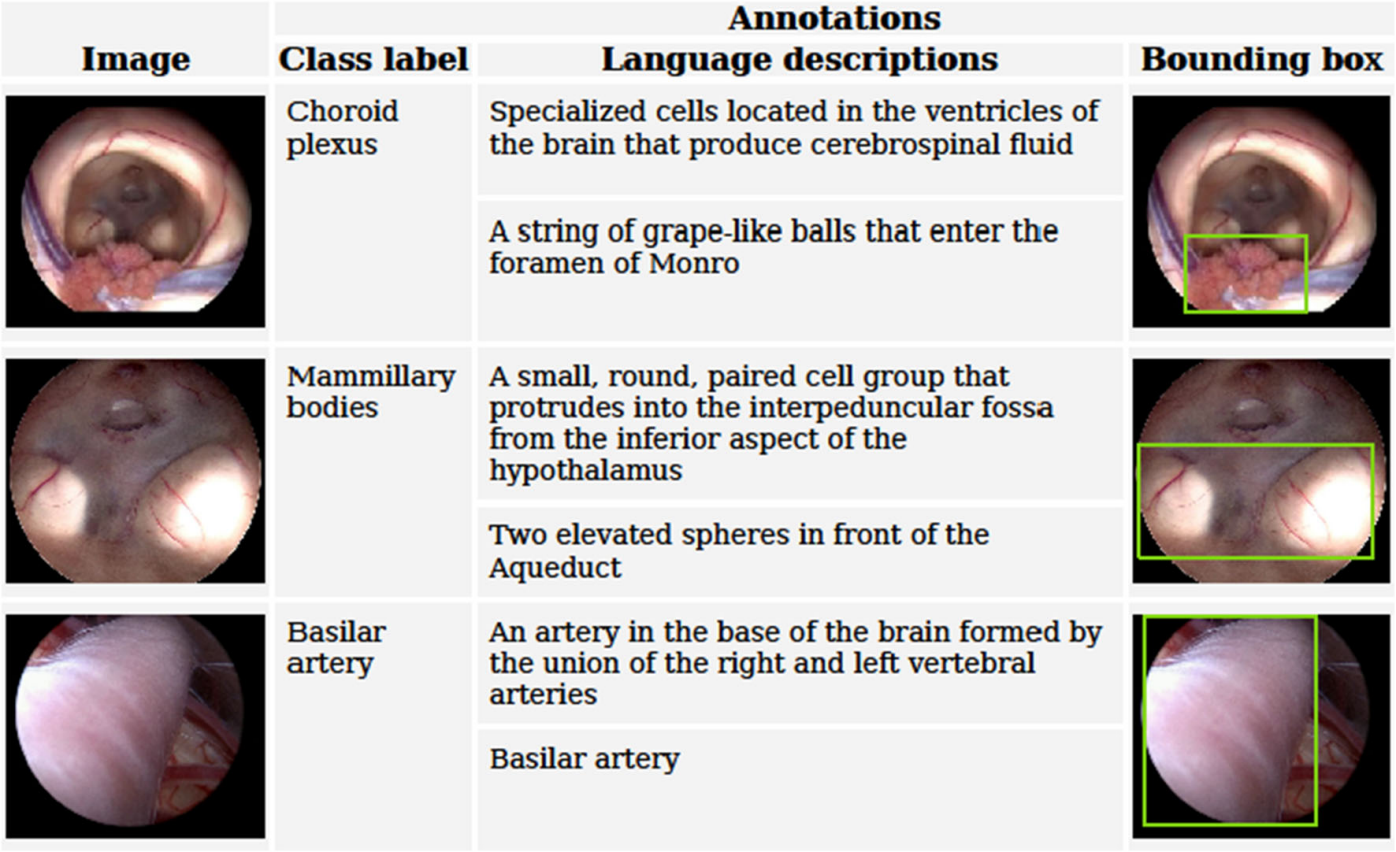
**Fig. 1** Representative sample dataset with annotated medical images, including organ labels, language descriptions, and bounding box annotations for anatomical structures

The original article has been corrected.

